# Comparative effectiveness and safety of pharmaceuticals assessed in observational studies compared with randomized controlled trials

**DOI:** 10.1186/s12916-021-02176-1

**Published:** 2021-12-06

**Authors:** Yoon Duk Hong, Jeroen P. Jansen, John Guerino, Marc L. Berger, William Crown, Wim G. Goettsch, C. Daniel Mullins, Richard J. Willke, Lucinda S. Orsini

**Affiliations:** 1 University of Maryland School of Pharmacy, Baltimore, MD USA; 2 Department of Clinical Pharmacy, School of Pharmacy, University of California–San Francisco, San Francisco, CA USA; 3 PrecisionHEOR, Oakland, CA USA; 4 Ipsos Healthcare, Parsippany, NJ USA; 5 Independent Consultant, New York, NY USA; 6 The Heller School for Social Policy and Management, Brandeis University, Waltham, MA USA; 7 Utrecht Centre of Pharmaceutical Policy, Division of Pharmacoepidemiology and Clinical Pharmacology, Utrecht University, Utrecht, The Netherlands; 8 National Health Care Institute, Diemen, The Netherlands; 9 ISPOR–The Professional Society for Health Economics and Outcomes Research, Lawrenceville, NJ USA; 10 COMPASS Pathways plc, London, UK

**Keywords:** Real-world evidence, Observational data, Pharmaceuticals

## Abstract

**Background:**

There have been ongoing efforts to understand when and how data from observational studies can be applied to clinical and regulatory decision making. The objective of this review was to assess the comparability of relative treatment effects of pharmaceuticals from observational studies and randomized controlled trials (RCTs).

**Methods:**

We searched PubMed and Embase for systematic literature reviews published between January 1, 1990, and January 31, 2020, that reported relative treatment effects of pharmaceuticals from both observational studies and RCTs. We extracted pooled relative effect estimates from observational studies and RCTs for each outcome, intervention-comparator, or indication assessed in the reviews. We calculated the ratio of the relative effect estimate from observational studies over that from RCTs, along with the corresponding 95% confidence interval (CI) for each pair of pooled RCT and observational study estimates, and we evaluated the consistency in relative treatment effects.

**Results:**

Thirty systematic reviews across 7 therapeutic areas were identified from the literature. We analyzed 74 pairs of pooled relative effect estimates from RCTs and observational studies from 29 reviews. There was no statistically significant difference (based on the 95% CI) in relative effect estimates between RCTs and observational studies in 79.7% of pairs. There was an extreme difference (ratio < 0.7 or > 1.43) in 43.2% of pairs, and, in 17.6% of pairs, there was a significant difference and the estimates pointed in opposite directions.

**Conclusions:**

Overall, our review shows that while there is no significant difference in the relative risk ratios between the majority of RCTs and observational studies compared, there is significant variation in about 20% of comparisons. The source of this variation should be the subject of further inquiry to elucidate how much of the variation is due to differences in patient populations versus biased estimates arising from issues with study design or analytical/statistical methods.

**Supplementary Information:**

The online version contains supplementary material available at 10.1186/s12916-021-02176-1.

## Background

Health care decision makers, particularly regulators but also health technology assessment agencies, have depended upon evidence from randomized clinical trials (RCTs) to assess drug effectiveness and to make comparisons among treatment options. Widespread adoption of the RCT was the hallmark of progress in clinical research in the twentieth century and accelerated the development and approval of new therapeutics; confidence in RCTs derived from their experimental nature, designs to minimize bias, rigorous data quality, and analytic approaches that supported causal inference.

In the last 30 years, we have witnessed an explosion of observational real-world data (RWD) and evidence (RWE) derived from RWD that has supplemented our understanding of the benefits and risks of treatments in broader populations of patients. RWE has been largely leveraged by regulators to assess the safety of marketed products and for new drug approvals when RCTs are infeasible, such as in rare diseases, oncology, or for long-term adverse effects. RCTs often do not have sufficient sample size to detect rare adverse events or long enough follow-up to detect long-term adverse effects. In such cases, regulatory decisions are often supplemented by RWE. However, leveraging of RWE has been much more slowly embraced in comparison to the adoption of RCTs for a variety of reasons. Imputation of causality is less certain in the absence of randomization and RWD can be much sparser and often requires extensive curation before it can be analyzed. Thus, skepticism about the robustness of observational RWD studies has made decision makers cautious in relying solely upon it to render judgments about the availability and appropriate use of new therapeutics, particularly by regulatory bodies.

Moreover, observational studies examining the effectiveness of treatments in similar populations have not always provided results consistent with RCTs. Despite many studies finding similar treatment effect estimates from RCTs and RWD analyses [1–3], other analyses have documented wide variation in results from RWD analyses within the same therapeutic areas [4], including analyses using propensity score-based methods [5]. Nonetheless, public interest has grown in the routine leveraging of RWD to promote the creation of a learning healthcare system, and regulatory bodies and other decision makers are exploring ways to expand their use of RWE. This is partly due to increasing acknowledgement of the value of RWE, such as its ability to better reflect actual environments in which the interventions are used.

One promising approach to understanding the sources of variability between RCT and observational study results is to compare estimates obtained from RWD analyses that attempt to emulate the eligibility criteria, endpoints, and other features of trials as closely as possible. A small number of RWD analyses have generated findings similar to previous RCTs [6, 7], and the findings of other RWD analyses have been consistent with subsequent RCTs [8]. In a small number of cases, RCTs and RWD studies have been published simultaneously [9]. This has the advantage of not knowing the RCT estimate when conducting the RWD study. There have been disagreements between observational RWD analyses and RCTs that were based upon avoidable errors in the RWD analysis design [7, 10]. This has led to a focus on the importance of research design in observational RWD analyses attempting to draw causal inferences regarding treatment effects [11–13].Emulation studies can improve understanding of when observational studies may reliably generate results consistent with RCTs; however, not all RCTs can be feasibly emulated using RWD due to limitations in observational datasets. Existing sources of observational data, such as health insurance claims and electronic health records (EHRs), may not routinely capture the intervention, indication, inclusion and exclusion criteria, and/or endpoints used in RCTs [14].

The objective of this paper is to provide further evidence on the comparability of RCTs and observational studies when the latter use a range of study designs and were not designed to emulate RCTs. We aim to quantify the extent of the difference in treatment effect estimates between RCTs and observational studies. We go beyond previous comparisons of RCTs and observational studies, with a focus purely on pharmaceuticals, and provide a systematic landscape review of the (in)consistency between RCT and observational study treatment effect estimates. The reasons for the variation in relative treatment effects are not assessed in this review but should be the subject of further study.

## Methods

### Eligibility criteria

#### Inclusion criteria

- Study design:
  - Published systematic literature reviews designed to compare relative treatment effects from observational studies with the corresponding effects from RCTs; *or*
  - Published systematic literature reviews that reported subgroup analyses stratified by RCT and observational study design; *and*
  - Observational studies included in these reviews have to be retrospective or prospective cohort studies, or case-control studies
- Population: Human subjects
- Intervention(s) and comparator(s): Any active or placebo-controlled pharmaceutical or biopharmaceutical intervention
- Outcome(s):
  - Efficacy/effectiveness or safety outcomes
  - Pooled relative treatment effect estimates for both observational studies and RCTs

#### Exclusion criteria

- Systematic reviews that compared absolute outcomes, such as event rates, between non-comparative observational studies and RCTs
- Non-pharmaceutical-based studies, e.g., surgical procedures, traditional medicine, vitamin/herbal supplements, etc.
- Non-English language
- Abstracts or conference proceedings

### Search strategy

We searched PubMed and Embase to identify relevant systematic literature reviews published between January 1, 1990, and January 31, 2020. Anglemeyer et al.’s search strategy [1] was used as a template to develop the search strategy, which included a wide range of MeSH terms and relevant keywords. We updated Anglemeyer et al.’s systematic review hedge and used the more recent CADTH systematic review/meta-analysis hedge, created in 2016, in both PubMed and Embase [15]. We restricted our search to focus on pharmaceuticals only. PubMed and Embase were searched for the following concepts: pharmaceuticals, study methodology, and comparisons (filters: Humans and English language). The PubMed search strategy which was adapted for use in Embase can be found in Additional File 1.

### Study selection

After removing duplicate references, three authors (JG, YH and LO) screened the titles and abstracts to identify relevant reviews. Once complete, LO verified the screening for accuracy. Following the title and abstract screen, full text articles were obtained for all potentially relevant reviews. Full text articles were then assessed to determine if they meet the selection criteria for final inclusion in the review.

### Data extraction

A pilot extraction was first done by two authors (JG and YH) on a sample of three articles using a standardized extraction table. This was done to test the standardized extraction table and to ensure consistency between the authors performing the data extraction. JG and YH then independently extracted information from each review using the standardized extraction table. A third author (LO) verified the extraction for accuracy and identified any discrepancies. These discrepancies were discussed until resolved.

We focused on primary outcomes reported in the reviews and extracted information summarizing the scope of each of the identified systematic reviews. Extracted information included the following: review objective, population, disease/therapeutic area, interventions, outcome(s), number of included RCTs and observational studies, pooled relative treatment effect estimates for RCTs and observational studies along with the 95% confidence intervals (95% CI), and measures of heterogeneity.

### Analysis

Based on the extracted information, we calculated the ratio of the relative treatment effect estimate from observational studies over the relative treatment effect estimate from RCTs (e.g., RR_obs_/RR_rct_), along with the corresponding 95% CI obtained via a Monte Carlo simulation for each pair of pooled RCT and observational study estimates. Outcomes for which the relative treatment effect was not expressed with a relative risk (RR), odds ratio (OR), or hazard ratio (HR) were excluded from our analysis.

We expressed differences in pooled effect estimates with the following measures: ratios that were < 1, > 1, or = 1, ratios indicating an “extreme difference” (< 0.70 or > 1.43) [16] and absence of an extreme difference. We evaluated (in)consistency between pooled RCT and observational study estimates with the following measures: presence of opposite direction of effect, RCT effect estimate outside the 95% CI of the observational study estimate, and vice versa, statistically significant difference between RCT and observational study estimates, and statistically significant difference along with opposite direction of effect. Statistically significant difference was determined by examining the 95% CI of the ratio of the relative treatment effect estimates from observational studies and RCTs derived from the Monte Carlo simulation. We examined differences in relative effect measures from observational studies and RCTs by outcome type and therapeutic area.

To test the robustness of our findings, we conducted two sensitivity analyses. As some reviews assessed more than one endpoint and contributed more than one pair of pooled relative treatment effects from RCTs and observational studies to our analysis, we repeated the analysis with one endpoint per review, i.e., a single pair of pooled relative treatment effects from RCTs and observational studies from each review, selecting the most frequently used endpoints for inclusion whenever possible. Additionally, as some studies were included in more than one review, we repeated the analysis ensuring that there is no overlap of data between the included reviews, i.e., ensuring that each study was included in only one review included in our analysis. Details on the sensitivity analyses are included in Additional File 2. All analyses were conducted using RStudio, version 1.3.1073 (©2009-2020 RStudio, PBC).

## Results

### Literature search

**Fig. 1:**
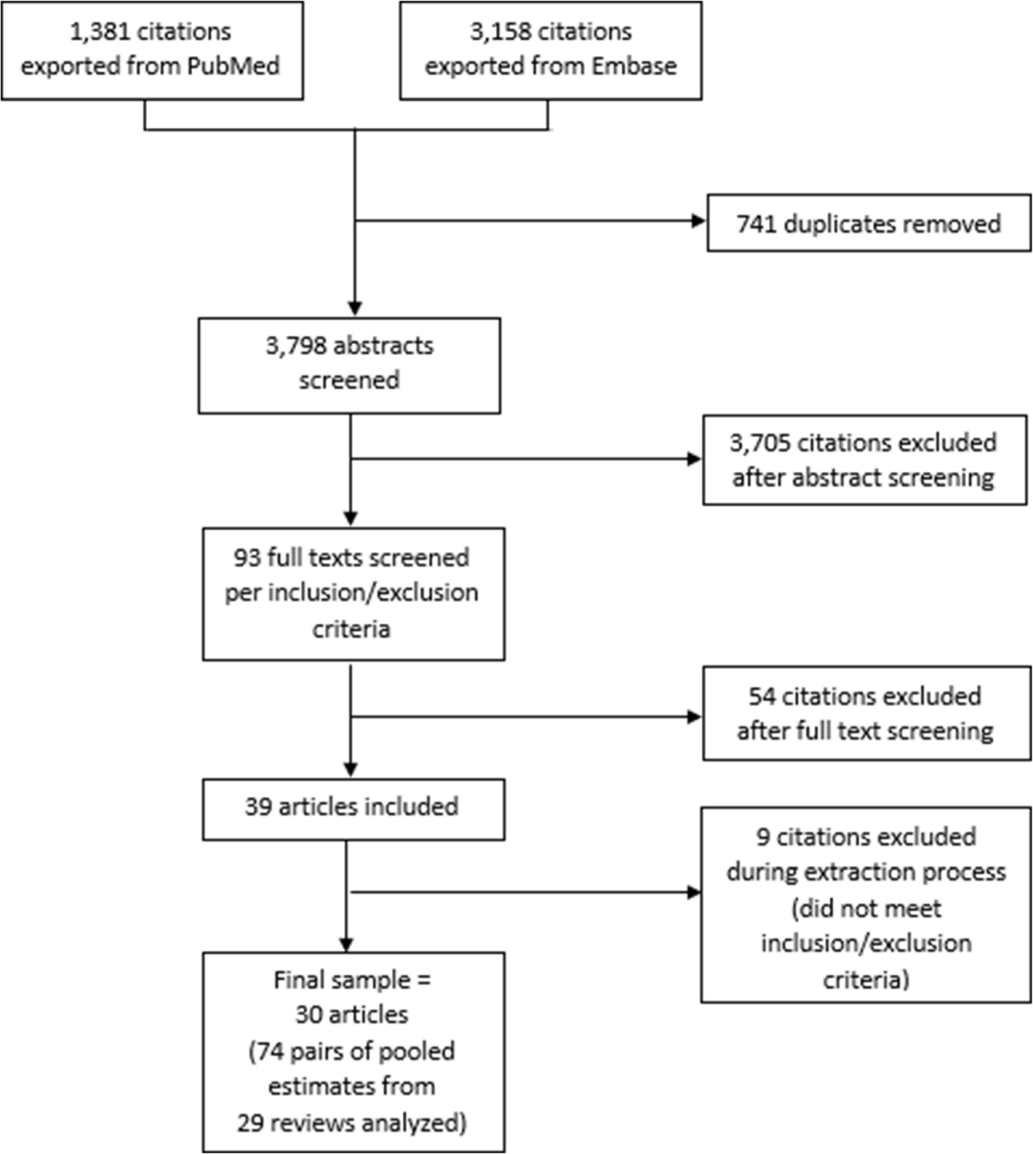
Diagram depicting literature screening process

Our search on PubMed and Embase yielded 3798 unique citations after removing duplicates. After screening titles and abstracts, we identified 93 full text articles for further review. Of these, 30 reviews met our inclusion criteria (Fig. 1).

### Included systematic reviews

The characteristics of the included reviews and the pairs of pooled relative treatment effects from RCTs and observational studies reported in the reviews are summarized in Table 1. Thirty systematic reviews across 7 therapeutic areas (cardiovascular disease [15/30], infectious disease [6/30], oncology [3/30], mental health [2/30], immune-inflammatory [1/30], metabolic disease [1/30], and other [2/30]) were identified from the literature. These reviews included 519 RCTs and observational studies and provided 79 pairs of pooled relative treatment effects from RCTs and observational studies across multiple interventions, comparators, and outcomes. Five pairs were excluded from our assessment because they concerned continuous outcomes (*n* = 1) or no pooled effect estimate was reported for observational studies (*n* = 4). As a result, 74 pairs of pooled relative treatment effects from RCTs and observational studies from 29 reviews were available for assessment of consistency.

**Table 1:** Characteristics of included reviews

Review	Disease	Treatment	Comparator	Endpoint	Number of RCTs	Number of Observational Studies	RCT pooled effect estimate (95% CI)	Observational pooled effect estimate (95% CI)
Abuzaid (2018) [17]	Severe aortic stenosis	Dual anti-platelet therapy (DAPT)	Single anti-platelet therapy (SAPT)	30-day all-cause mortality	3	7	RR 1.2 (0.5–2.89)	RR 1.19 (0.74–1.91)
Abuzaid (2018) [17]	Severe aortic stenosis	DAPT	SAPT	Longest reported all-cause mortality	3	7	RR 1.14 (0.54–2.42)	RR 1.06 (0.52–2.18)
Abuzaid (2018) [17]	Severe aortic stenosis	DAPT	SAPT	Major bleeding	3	7	RR 1.74 (0.52–5.82)	RR 2.23 (1.36–3.65)
Agarwal (2019) [18]	Acute coronary syndrome (ACS), coronary artery disease (CAD)	Dual therapy	Triple therapy	Major bleeding	3	3	RR 0.53 (0.38–0.76)	RR 0.88 (0.46–1.67)
Agarwal (2018) [19]	CAD	DAPT	Aspirin	Primary outcome: mid- to long-term (> 30 days) composite of myocardial infarction (MI), stroke, or death	8	4	RR 0.43 (0.17–1.11)	RR 0.85 (0.72–1.01)
An (2019) [20]	Severe aortic stenosis	Antiplatelet	Anticoagulation	Mortality	2	5	RR 0.82 (0.33–2.03)	RR 0.47 (0.18–1.22)
An (2019) [20]	Severe aortic stenosis	Antiplatelet	Anticoagulation	Stroke/transient ischemic attack (TIA)	2	5	RR 0.9 (0.35–2.33)	RR 0.57 (0.31–1.03)
An (2019) [20]	Severe aortic stenosis	Antiplatelet	Anticoagulation	Thromboembolic events	2	5	RR 1.13 (0.51–2.49)	RR 0.71 (0.38–1.32)
An (2019) [20]	Severe aortic stenosis	Antiplatelet	Anticoagulation	Bleeding	2	5	RR 0.34 (0.11–1.04)	RR 0.34 (0.2–0.58)
Chien (2020)* [21]	Multi-drug resistant gram-negative bacteria (MDR-GNB) infections	Colistin	Other antibiotics	Colistin-associated acute kidney injury (CA-AKI)	1	19	OR 2.75 (0.43–17.49)	Not reported
Chien (2020) [21]	MDR-GNB infections	Colistin monotherapy	Colistin combination therapy	Acute kidney injury (AKI)	3	6	OR 1.77 (1.17–2.66)	OR 1.15 (0.76–1.76)
Chopra (2012)* [22]	Pneumonia	Statin therapy	No statin therapy	Unadjusted all-cause mortality following an episode of pneumonia	1	9	OR 0.84 (0.32–2.18)	Not reported
Chopra (2012) [22]	Pneumonia	Statin therapy	No statin therapy	Adjusted all-cause mortality following an episode of pneumonia	1	11	OR 0.84 (0.32–2.18)	OR 0.66 (0.55–0.79)
Desai (2016) [23]	Ankylosing spondylitis, inflammatory bowel diseases, juvenile idiopathic arthritis, plaque psoriasis, psoriatic arthritis, and rheumatoid arthritis	Adalimumab	Etanercept	Discontinuation due to adverse events	1	3	RR 0.83 (0.39–1.78)	Adjusted HR 1.67 (1.26–2.22)
Desai (2016) [23]	Ankylosing spondylitis, inflammatory bowel diseases, juvenile idiopathic arthritis, plaque psoriasis, psoriatic arthritis, rheumatoid arthritis	Adalimumab	Infliximab	Discontinuation due to adverse events	1	5	RR 6.17 (0.78–48.71)	Adjusted HR 0.57 (0.46–0.7)
Gandhi (2015) [24]	Aortic stenosis	DAPT	Mono-antiplatelet therapy (MAPT).	Combined end point of 30-day major stroke, spontaneous MI, all-cause mortality, and combined lethal and major bleeding	2	2	OR 0.98 (0.46–2.11)	OR 3.02 (1.91–4.76)
Ge (2018) [25]	Atrial fibrillation	Novel oral anticoagulants (NOACs)	Vitamin K antagonists (VKAs)	Major bleeding events (Fixed effects model)	4	25	OR 0.3 (0.14–0.62)	OR 0.68 (0.48–0.95)
Ge (2018) [25]	Atrial fibrillation	NOACs	VKAs	Thromboembolic events (Fixed effects model)	4	25	OR 0.14 (0.01–1.3)	OR 0.91 (0.49–1.67)
Heffernan (2020) [26]	Serious infections	β-lactam/aminoglycoside combination therapy	β-lactam monotherapy	All-cause mortality	2	4	OR 3.18 (0.79–12.73)	OR 0.79 (0.64–0.99)
Ho (2013) [27]	Kidney disease (kidney transplant)	Once daily tacrolimus	Twice daily tacrolimus	Biopsy-proven acute rejection (RCT: **at 6 months**; Observational: mean follow-up ranges from 3 months to 672 months)	4	5	RR 1.18 (0.82–1.68)	RR 0.83 (0.39–1.78)
Ho (2013) [27]	Kidney disease (kidney transplant)	Once daily tacrolimus	Twice daily tacrolimus	Biopsy-proven acute rejection (RCT: **at 12 months** Observational: mean follow-up ranges from 3 months to 672 months)	2	5	RR 1.24 (0.93–1.65)	RR 0.83 (0.39–1.78)
Ho (2013) [27]	Kidney disease (kidney transplant)	RCT: twice daily tacrolimus Observational: once daily tacrolimus	RCT: once daily tacrolimus Observational: twice daily tacrolimus	Patient survival (RCT: **at 6 months** Observational: mean follow-up ranges from 3.5 months to 12 months)	2	2	RR 1.03 (1–1.06)	RR 1.02 (0.94–1.1)
Ho (2013) [27]	Kidney disease (kidney transplant)	RCT: twice daily tacrolimus Observational: once daily tacrolimus	RCT: once daily tacrolimus Observational: twice daily tacrolimus	Patient survival (RCT: **at 12 months** Observational: mean follow-up ranges from 3.5 months to 12 months)	3	2	RR 0.99 (0.97–1.02)	RR 1.02 (0.94–1.1)
Khan (2019) [28]	CAD	Proton pump inhibitor (PPI)	No PPI	All-cause mortality	3	24	RR 1.35 (0.56–3.23)	RR 1.25 (1.11–1.41)
Kirson (2013)* [29]	Schizophrenia	Depot antipsychotics	Oral antipsychotics	Varies across studies: hospitalization, relapse, discontinuation	5	8	RR 0.89 (0.64–1.22)	Not reported
Land (2017) [30]	Psychiatric illnesses	Clozapine	Control drugs (other antipsychotics)	Hospitalization	3	19	RR 0.62 (0.41–0.94)	RR 0.75 (0.69–0.81)
Li (2016) [31]	Diabetes	Dipeptidyl peptidase-4 (DPP-4) inhibitors	RCT: control Observational: sulfonylurea (SU)	Heart failure	38	1	OR 0.97 (0.61–1.56)	Unadjusted OR 0.88 (0.22–3.48)
Li (2016) [31]	Diabetes	DPP-4 inhibitors	RCT: control Observational: SU	Heart failure	38	1	OR 0.97 (0.61–1.56)	Adjusted HR 1.10 (1.04–1.17)
Li (2016) [31]	Diabetes	RCT: DPP-4 inhibitors Observational: sitagliptin	RCT: control Observational: SU	Heart failure	38	1	OR 0.97 (0.61–1.56)	Unadjusted OR 0.39 (0.02–6.26)
Li (2016) [31]	Diabetes	RCT: DPP-4 inhibitors Observational: sitagliptin	RCT: control Observational: no sitagliptin use	Heart failure	38	1	OR 0.97 (0.61–1.56)	Adjusted OR 0.75 (0.38–1.46)
Li (2016) [31]	Diabetes	DPP-4 inhibitors	RCT: control Observational: active control	Hospital admission for heart failure	5	6	OR 1.13 (1.00–1.26)	Adjusted OR 0.85 (0.74–0.97)
Li (2016) [31]	Diabetes	DPP-4 inhibitors	RCT: control Observational: SU	Hospital admission for heart failure	5	3	OR 1.13 (1.00–1.26)	Adjusted HR 0.84 (0.74–0.96)
Li (2016) [31]	Diabetes	DPP-4 inhibitors	RCT: control Observational: pioglitazone	Hospital admission for heart failure	5	2	OR 1.13 (1.00–1.26)	Adjusted HR 0.67 (0.57–0.78)
Li (2016) [31]	Diabetes	DPP-4 inhibitors	RCT: control Observational: other oral antidiabetics	Hospital admission for heart failure	5	1	OR 1.13 (1.00–1.26)	Adjusted OR 0.88 (0.63–1.22)
Li (2016) [31]	Diabetes	DPP-4 inhibitors	Control	Hospital admission for heart failure	5	1	OR 1.13 (1.00–1.26)	Adjusted HR 0.58 (0.38–0.88)
Li (2016) [31]	Diabetes	RCT: DPP-4 inhibitors Observational: sitagliptin	RCT: control Observational: no sitagliptin use	Hospital admission for heart failure	5	2	OR 1.13 (1.00–1.26)	Adjusted OR 1.41 (0.95–2.09)
Li (2016) [31]	Diabetes	RCT: DPP-4 inhibitors Observational: sitagliptin	RCT: control Observational: no sitagliptin use	Hospital admission for heart failure	5	1	OR 1.13 (1.00–1.26)	Adjusted HR 1.21 (1.04–1.42)
Li (2016) [31]	Diabetes	RCT: DPP-4 inhibitors Observational: sitagliptin	RCT: control Observational: no sitagliptin use	Hospital admission for heart failure	5	1	OR 1.13 (1.00–1.26)	Adjusted OR 1.84 (1.16–2.92)
Melloni (2015) [32]	Unstable angina/non–ST-segment–elevation myocardial infarction (UA/NSTEMI)	RCT: omeprazole Observational: any PPI	RCT: placebo Observational: no PPI	Composite ischemic endpoint at ≈ 1 year	1	20	HR 0.99 (0.68–1.44)	Adjusted HR 1.35 (1.18–1.54)
Melloni (2015) [32]	UA/NSTEMI	RCT: omeprazole Observational: any PPI	RCT: placebo Observational: no PPI	Nonfatal MI at ≈ 1 year	1	10	HR 0.92 (0.44–1.9)	HR 1.331 (1.146–1.547)
Miles (2019) [33]	Heart failure	Furosemide	Torsemide	All-cause mortality	5	3	OR 1.12 (0.7–1.8)	OR 0.97 (0.44–2.13)
Miles (2019) [33]	Heart failure	Furosemide	Torsemide	Heart failure readmissions	4	1	OR 2.04 (1.16–3.60)	OR 2.91 (0.78–10.91)
Miles (2019) [33]	Heart failure	Furosemide	Torsemide	New York Heart Association class improvement	7	2	OR 0.91 (0.61–1.35)	OR 0.65 (0.50–0.85)
Mongkhon (2019) [34]	Atrial fibrillation	OAC	Non-OAC	Risk of dementia	1	4	RR 1.31 (0.79–2.18)	RR 0.75 (0.67–0.83)
Mongkhon (2019) [34]	Atrial fibrillation	VKA	Non-VKA	Risk of dementia	1	4	RR 1.31 (0.79–2.18)	RR 0.71 (0.68–0.74)
Raheja (2018) [35]	Aortic stenosis	DAPT	SAPT	All-cause mortality	3	2	RR 1.07 (0.48–2.41)	RR 1.34 (0.51–3.48)
Raheja (2018) [35]	Aortic stenosis	DAPT	SAPT	Stroke or TIA	3	2	RR 0.93 (0.28–3.06)	RR 1.25 (0.32–4.92)
Raheja (2018) [35]	Aortic stenosis	DAPT	SAPT	MI	3	2	RR 3.62 (0.60–21.76)	RR 1.18 (0.14–9.98)
Raheja (2018) [35]	Aortic stenosis	DAPT	SAPT	Major/life-threatening bleeding	3	3	RR 1.75 (0.88–3.50)	RR 3.24 (1.82–5.75)
Ramjan (2014) [36]	HIV	Fixed-dose combination (FDC) antiretroviral therapy (ART)	Separate tablet regimens	Virological suppression	4	2	RR 1.04 (0.98–1.10)	RR 1.07 (0.97–1.18)
Ramjan (2014) [36]	HIV	FDC ART	Separate tablet regimens	Adherence to ART	5	2	RR 1.1 (0.98–1.22)	RR 1.17 (1.07–1.28)
Shi (2014) [37]	Liver cancer	Statins	Placebo/non-use	Liver cancer	1	11	RR 1.06 (0.66–1.71)	RR 0.57 (0.50–0.64)
Teo (2014) [38]	Acute infections	Prolonged infusion, which was defined as administration of either extended infusion or continuous infusion of beta-lactam antibiotics	Identical beta-lactams that were administered as intermittent boluses (20–60 min infusion) according to the manufacturer’s package insert	All-cause in-hospital mortality	10	9	RR 0.83 (0.57–1.21)	RR 0.57 (0.43–0.76)
Teo (2014) [38]	Acute infections	Prolonged infusion, which was defined as administration of either extended infusion or continuous infusion of beta-lactam antibiotics.	Identical beta-lactams that were administered as intermittent boluses (20–60 min infusion) according to the manufacturer’s package insert	Clinical success (cure or improvement)	14	5	RR 1.05 (0.99–1.12)	RR 1.34 (1.02–1.76)
Vinceti (2018) [39]	Cancer	Highest selenium exposure	Lowest selenium exposure	Total (any) cancer incidence	3	7	RR 1.01 (0.93–1.10)	OR 0.72 (0.55–0.93)
Vinceti (2018) [39]	Cancer	Highest selenium exposure	Lowest selenium exposure	Cancer mortality	1	7	RR 1.02 (0.80–1.30)	OR 0.76 (0.59–0.97)
Vinceti (2018) [39]	Colorectal cancer	Highest selenium exposure	Lowest selenium exposure	Colorectal cancer risk	2	1	RR 0.99 (0.69–1.43)	OR 0.80 (0.68–0.94)
Vinceti (2018) [39]	Lung cancer	Highest selenium exposure	Lowest selenium exposure	Lung cancer risk	2	5	RR 1.16 (0.89–1.50)	OR 0.74 (0.43–1.28)
Vinceti (2018) [39]	Breast cancer	Highest selenium exposure	Lowest selenium exposure	Breast cancer risk	1	8	RR 2.04 (0.44–9.55)	OR 1.09 (0.87–1.37)
Vinceti (2018) [39]	Bladder cancer	Highest selenium exposure	Lowest selenium exposure	Bladder cancer risk	2	2	RR 1.07 (0.76–1.52)	OR 0.65 (0.46–0.92)
Vinceti (2018) [39]	Prostate cancer	Highest selenium exposure	Lowest selenium exposure	Prostate cancer risk	4	21	RR 1.01 (0.90–1.14)	OR 0.84 (0.75–0.95)
Wang (2019) [40]	Pneumonia	PPI	No PPI	Pneumonia	10	48	OR 1.13 (0.71–1.78)	OR 1.45 (1.32–1.59)
Wat (2019) [41]	Traumatic brain injury (TBI)	Antiepileptic drugs	Placebo/no treatment	Early seizures after TBI	3	6	RR 0.58 (0.20–1.72)	RR 0.42 (0.29–0.62)
Wong (2017)* [42]	Coronary heart disease/CAD	Macrolides	Placebo/no treatment	Short-term primary outcome (defined as cardiac mortality, cardiovascular mortality, sudden death, cardiac arrest, all-cause mortality, or composite outcomes including death and/or other cardiovascular events or procedures)	5	15	RR 0.99 (0.74–1.34)	Not reported
Wong (2017) [42]	Coronary heart disease/CAD	Macrolides	Placebo/no treatment	Long term primary outcome (defined as cardiac mortality, cardiovascular mortality, sudden death, cardiac arrest, all-cause mortality, or composite outcomes including death and/or other cardiovascular events or procedures)	14	8	RR 1.03 (0.96–1.10)	RR 1.05 (0.91–1.22)
Yang (2019)* [43]	Cancer	Epoetin alfa biosimilar drugs	Epoetin alfa drugs	Mean of hemoglobin increase	1	4	WMD -0.02 (− 0.38–0.34)	WMD 0.07 (− 0.12–0.25)
Yang (2019) [43]	Cancer	Epoetin alfa biosimilar drugs	Epoetin alfa drugs	Hemoglobin response	1	1	RR 1.09 (0.86–1.38)	RR 1.18 (0.87–1.60)
Yang (2019) [43]	Breast cancer	Granulocyte colony-stimulating factor (G-CSF) biosimilar drugs	G-CSF drugs	Febrile neutropenia in cycle 1	5	3	RR 1.14 (0.80–1.63)	RR 1.36 (0.84–2.23)
Yang (2019) [43]	NHL	G-CSF biosimilar drugs	G-CSF drugs	Febrile neutropenia in cycle 1	1	1	RR 0.54 (0.20–1.46)	RR 0.87 (0.20–3.85)
Yang (2019) [43]	Cancer	G-CSF biosimilar drugs (filgrastim biosimilars)	G-CSF drugs	Bone pain	4	4	RR 0.90 (0.78–1.05)	RR 0.86 (0.59–1.24)
Yu (2018) [44]	Non-cardiac vascular disease	Statins	Placebo/no statin treatment	All-cause mortality	3	6	OR 0.62 (0.41–0.92)	OR 0.65 (0.48–0.88)
Yu (2018) [44]	Non-cardiac vascular disease	Statins	Placebo/no statin treatment	Primary patency	1	10	OR 0.39 (0.09–1.65)	OR 0.77 (0.59–0.99)
Yu (2018) [44]	Non-cardiac vascular disease	Statins	Placebo/no statin treatment	Amputation	1	10	OR 0.47 (0.07–2.94)	OR 0.64 (0.50–0.83)
Yu (2018) [44]	Non-cardiac vascular disease	Statins	Placebo/no statin treatment	Cardiovascular events	3	2	OR 0.55 (0.36–0.83)	OR 0.87 (0.16–4.60)
Zhang (2019) [45]	Atrial fibrillation	NOAC	Non-NOAC therapy	Renal impairment	11	3	HR 0.82 (0.71–0.93)	HR 0.64 (0.58–0.69)
Zhao (2018) [46]	CAD	DAPT	SAPT	Any bleeding events	5	8	RR 1.25 (0.98–1.59)	RR 0.87 (0.76–1.01)
Zhao (2018) [46]	CAD	DAPT	SAPT	Minor bleeding events	4	3	RR 1.15 (0.73–1.81)	RR 0.84 (0.37–1.93)
Zhao (2018) [46]	CAD	DAPT	SAPT	Major bleeding events	5	6	RR 1.28 (0.95–1.71)	RR 0.99 (0.66–1.51)
Zhao (2018) [46]	CAD	DAPT	SAPT	Major bleeding events during hospitalization (random effects model)	3	3	RR 1.27 (0.91–1.78)	RR 0.50 (0.12–2.09)

*Not included in the analysis

### Ratio of relative effect measures from observational studies and RCTs

**Fig. 2:**
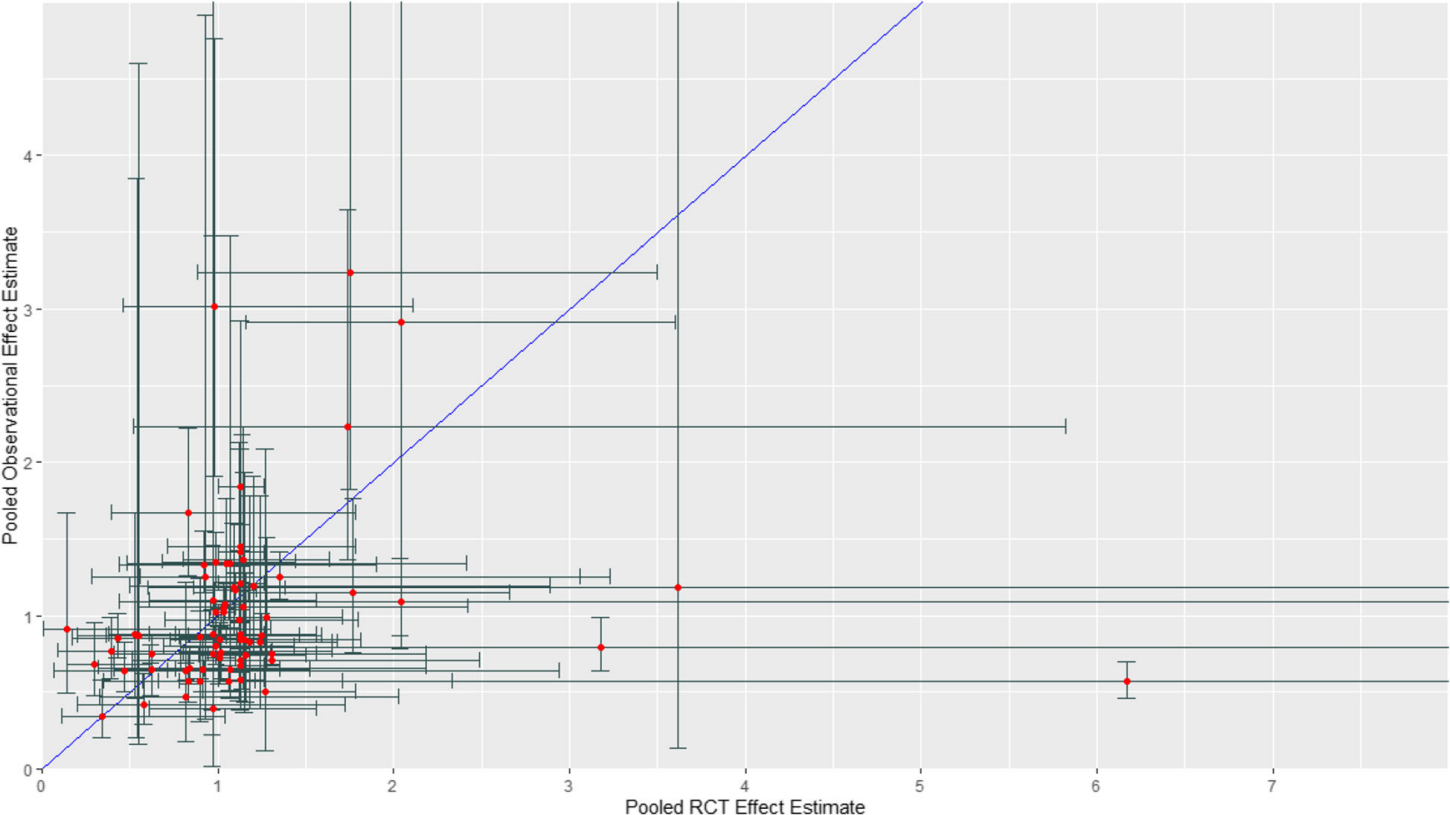
Relative effect measures (RR, OR, HR) from observational studies (y-axis) versus corresponding relative effect measures from randomized controlled trials (x-axis) across 74 pairs of pooled relative treatment effects

Figure 2 presents the scatterplot of relative effect measures from observational studies and RCTs across the 74 pairs of pooled relative treatment effects with the 95% CI bars. The ratio of the relative effect measure from observational studies over the corresponding relative effect measure from RCTs ranged from 0.09 to 6.50 (median = 0.92, interquartile range = 0.69–1.27). The ratio was greater than 1, i.e., the relative effect was larger in observational studies in 31 of the 74 pairs (41.9%). The ratio was less than 1, i.e., the relative effect was larger in RCTs in 42 of the 74 pairs (56.8%), and the ratio was equal to 1 in one of the 74 pairs (1.4%). The ratio was greater than 1.43 in 12 of the 74 pairs (16.2%) and less than 0.7 in 20 of the 74 pairs (27.0%) indicating an extreme difference. There was an absence of an extreme difference (0.7 ≤ ratio ≤ 1.43) in 42 of the 74 pairs (56.8%; Table 2). Sensitivity analyses including only one endpoint from each review and ensuring no overlap of data between the included reviews resulted in similar findings (Table 2). Scatterplots of relative effect measures from observational studies and RCTs by outcome type and therapeutic area can be found in Additional File 3: Figures S1 and S2.

**Table 2:** Ratio of relative effect measures from observational studies and relative effect measures from RCTs (e.g., RR_obs_/RR_rct_): (a) among 74 pairs of pooled estimates, (b) with only one endpoint per review included, and (c) with studies reported in multiple reviews excluded

	Full sample		One endpoint per review		Studies reported in multiple reviews excluded	
	Proportion	%	Proportion	%	Proportion	%
Ratio > 1*^a^	31/74	41.9	12/29	41.4	24/65	36.9
Ratio < 1*^b^	42/74	56.8	17/29	58.6	40/65	61.5
Ratio = 1*	1/74	1.4	0/29	0.0	1/65	1.5
Extreme difference (ratio > 1.43)	12/74	16.2	5/29	17.2	8/65	12.3
Extreme difference (ratio < 0.7)	20/74	27.0	8/29	27.6	19/65	29.2
Absence of an extreme difference (0.7 ≤ ratio ≤ 1.43)	42/74	56.8	16/29	55.2	38/65	58.5

*Does not account for direction of effect

^*a*^Relative effect larger in observational studies

^*b*^Relative effect larger in RCTs

### Consistency of relative effect measures from observational studies and RCTs

In 30 of the 74 pairs (40.5%), effect estimates from observational studies and RCTs pointed in opposite directions of effect. The RCT point estimate was outside the 95% CI of the observational study in 35 of the 74 pairs (47.3%) and the observational study point estimate was outside the 95% CI of the RCT in 27 of the 74 pairs (36.5%). There was a statistically significant difference between relative effect estimates from observational studies and RCTs in 15 of the 74 pairs (20.3%). In 13 of the 74 pairs (17.6%), there was a statistically significant difference and the effect estimates of observational studies and RCTs pointed in opposite directions (Table 3). The results remained fairly consistent when the sensitivity analyses were conducted (Table 3).

**Table 3:** Consistency of relative effect measures from observational studies and relative effect measures from RCTs: (a) among 74 pairs of pooled estimates, (b) with only one endpoint per review included, and (c) with studies reported in multiple reviews excluded

	Full sample		One endpoint per review		Studies reported in multiple reviews excluded	
	Proportion	%	Proportion	%	Proportion	%
Effect estimates of observational studies and RCTs in opposite directions	30/74	40.5	11/29	37.9	26/65	40.0
RCT effect estimate outside the observational study 95% CI	35/74	47.3	17/29	58.6	29/65	44.6
Observational effect estimate outside the RCT 95% CI	27/74	36.5	11/29	37.9	25/65	38.5
Statistically significant difference	15/74	20.3	7/29	24.1	14/65	21.5
Statistically significant difference and effect estimates of observational studies and RCTs in opposite directions	13/74	17.6	6/29	20.7	12/65	18.5

## Discussion

Our analysis of 29 reviews comparing results of RCTs and observational studies of pharmaceuticals showed, on average, no significant differences in their relative risk ratios across all studies, but also considerable study-by-study variability. The median ratio of the relative effect measure from observational studies to RCTs was 0.92, indicating just slightly lower effectiveness/safety estimates in observational studies than corresponding RCTs. This is in fact somewhat higher than the 0.80 ratio recently found in meta-research comparing effect estimates of randomized clinical trials that use routinely collected data (i.e., from traditional observational study sources such as registries, electronic health records, or administrative claims) for outcome ascertainment with traditional trials not using routinely collected data [47]. However, whether judging by the frequency of “extreme” differences (43.2%) or statistically significant differences in opposite directions (17.6%), one could not claim that observational study results consistently replicated RCT results on a study-by-study basis in our sample.

There are a number of reasons that any given observational study result may not replicate an RCT comparing the same treatments. First, it may not have been the intent of the observational study researchers to match a specific clinical trial—they may have intentionally studied a different treatment population, setting, or protocol in order to complement or test the RCT findings. In such cases, there would be variation in effect estimates due to estimating a different causal effect. Even if the researcher does attempt to match a specific RCT, the data may not have been available to closely match it, since patient histories, test results, etc., used for RCT inclusion criteria may not be observed, or outcomes may not be captured the same way. Even given similar data, non-randomized studies have the potential for selection/channeling bias into treatment determined by factors unobservable in either type of study, and analytic attempts to correct for such confounding may have limited success. In some cases, treatment conditions may differ enough between the RCT and real-world practice that replication of results should not be expected, e.g., due to careful safety monitoring that affects subsequent treatment in RCTs. Finally, it is possible that other pharmacoepidemiologic principles, beyond the study design considerations we already mentioned, were violated in the individual RWD studies, which could have caused disagreement between their results and the RCTs. While variation in treatment effect estimates due to estimating a different causal effect in a different study population is expected and valid, biased estimates arising from issues with study design or analytical methods may be problematic.

Details in these reviews were typically insufficient to distinguish among these possible explanations, without detailed review of the individual studies, which we did not attempt here. However, some reviews did attempt to explain the differences they found. For example, in the review by Gandhi et al. (2015) [24], which compared dual-antiplatelet therapy (DAPT) to mono-antiplatelet therapy (MAPT) following transcatheter aortic valve implantation, there was a statistically significant difference in pooled relative treatment effect estimates from observational studies and RCTs. The primary outcome was more likely to occur in the DAPT group than in the MAPT group in the observational studies (OR 3.02; 95% CI 1.91–4.76); however, no statistically significant difference was found between DAPT and MAPT in the RCTs (OR 0.98; 95% CI 0.46–2.11). The authors explained that the RCTs (*n* = 2) and observational studies (*n* = 2) included in this review had variable patient inclusion/exclusion criteria and there were differences in the type of prosthetic aortic valve used, which may have introduced selection bias [24].

To allow for better use of individual observational studies to inform decision-making, their ability to replicate RCT results needs to become more reliable, and the “target trial” approach seems to be a path forward. Several systematic efforts using sophisticated observational data research designs to emulate multiple RCTs are underway [48, 49]. These efforts are intended to provide regulatory bodies and other decision makers with empirical evidence to support the development of a framework for assessing when and under what circumstances observational RWE can be used to support a wider range of regulatory decisions. RCT DUPLICATE is a collaboration between the Food and Drug Administration (FDA), Brigham and Women’s Hospital and Harvard Medical School Division of Pharmacoepidemiology, to replicate 30 completed Phase III or IV trials and to predict the results of seven ongoing Phase IV trials using Medicare and commercial claims data [50]. The RCT DUPLICATE team has recently reported results for its first 10 trials [51]. They report hazard ratio estimates within the 95% CI of the corresponding trial for 8 of 10 emulations.

The Multi-Regional Clinical Trials Center and OptumLabs are leading another effort called Observational Patient Evidence for Regulatory Approval and Understanding Disease (OPERAND) which extends the trial emulation activity and relaxes the inclusion/exclusion criteria of the trials to examine treatment effects in the broader patient population treated in routine care [52]. The FDA has also funded the Yale University-Mayo Clinic Center of Excellence in Regulatory Science and Innovation to predict the results of three to four ongoing safety trials using OptumLabs claims data [53].

It is important to understand that clinical trials emulation efforts are being conducted solely to improve understanding of when observational studies may be expected to produce robust results. Bartlett and colleagues [14] found that in a review of 220 clinical trials published in high impact medical journals in 2017, 15% could potentially be emulated using data available from medical claims or EHRs. For example, the inclusion/exclusion criteria for many oncology trials require data on genetic markers and progression free survival unavailable in EHRs. The estimate by Bartlett and colleagues may prove to be an underestimate as the ability to link different types of observational data continues to improve. Nevertheless, it is reasonable to assume that it is not possible to emulate most trials with existing observational datasets.

These efforts are critical to advance our understanding of the strengths and limitations of observational RWE, identifying issues with study design, endpoint definition, data quality, and analytical methodology that may impact the consistency of findings between RWE and RCTs. While much attention has focused on differences in study populations between observational studies and RCTs as the reason for the inconsistency in effect estimates, emerging evidence suggests that issues with study design (e.g., establishing time zero of exposure) may be equally if not more important [7]. Therefore, the results of these efforts will not provide definitive guidance to decision makers but they emphasize how even subtle differences in study design and endpoint definition can impact absolute estimates of treatment effect. Moreover, RWE studies are answering a different question than RCTs, i.e., “Does it work?” verses “Can it Work?” The former is important to a variety of stakeholders beyond regulators. Hence, they should not be expected to provide results identical to RCTs.

## Conclusions

In conclusion, although our review shows no average significant difference in the relative risk ratios between published RCTs and observational studies, there is substantial study-to-study variation. It was impractical to review all individual observational study designs and examine their potential biases, but future work should elucidate how much of the variation is due to differences in study populations versus biased estimates arising from issues with study design or analytical methods. As more target trial replication attempts are conducted and published, more systematic evidence will emerge on the reliability of this approach and on the potential for observational studies to more routinely inform healthcare decisions.

## Supplementary Information

**Additional File 1:.** Search Strategy

**Additional File 2:.** Sensitivity Analyses

**Additional File 3:.** Figures S1 & S2. Figure S1. Relative effect measures from observational studies versus corresponding relative effect measures from randomized controlled trials by outcome type. Figure S2. Relative effect measures from observational studies versus corresponding relative effect measures from randomized controlled trials by therapeutic area.

## Data Availability

The data analyzed in this study are included in this published article.

## Abbreviations

CI: Confidence interval
DAPT: Dual-antiplatelet therapy
EHR: Electronic health record
FDA: Food and Drug Administration
HR: Hazard ratio
MAPT: Mono-antiplatelet therapy
OPERAND: Observational Patient Evidence for Regulatory Approval and Understanding Disease
OR: Odds ratio
RCT: Randomized controlled trial
RWD: Real-world data
RWE: Real-world evidence
RR: Relative risk
