# Supplementary material for: Comparative effectiveness and safety of pharmaceuticals assessed in observational studies compared with randomized controlled trials
Source: BMC Med. 2021 Dec 6;19:307. doi: 10.1186/s12916-021-02176-1 (PMC8647453; doi:10.1186/s12916-021-02176-1)
Supplement: Supplementary file 1 — Additional File 1:. Search Strategy [file 12916_2021_2176_MOESM1_ESM.docx]

**Search Strategy:**

((”Observation“[mh] OR ”Cohort Studies“[mh] OR ”Longitudinal Studies“[mh] OR ”Retrospective Studies“[mh] OR ”Prospective Studies“[mh] OR observational[tiab] OR cohort*[tiab] OR crosssectional[tiab] OR crossectional[tiab] OR cross-sectional[tiab] OR cross sectional[tiab] OR longitudinal[tiab] OR causal inference*[tw] OR causality[tw] OR “instrumental variable”[tw] OR “structural model”[tw] OR practice-based[tw] OR propensity score*[tw] OR natural experiment*[tw] OR case-control[tw] OR before-after[tw] OR pre-post[tw] OR case-cohort[tw] OR case-crossover[tw] OR serial[tiab] OR nonexperimental[tiab] OR non-experimental[tiab] OR “nonrandomized”[tiab] OR “nonrandomised”[tiab] OR “non-randomised”[tiab] OR “nonrandomised”[tiab] OR “study designs”[tiab] OR “newcastle ottawa”[tiab] OR overestimat*[tiab] OR over-estimat*[tiab] OR bias[tiab] OR ”are needed“[tiab] OR (evidence[tiab] AND quality[tiab]))) AND (systematic[sb] OR meta-analysis[pt] OR meta-analysis as topic[mh] OR meta-analysis[mh] OR meta analy*[tw] OR metanaly*[tw] OR metaanaly*[tw] OR met analy*[tw] OR integrative research[tiab] OR integrative review*[tiab] OR integrative overview*[tiab] OR research integration*[tiab] OR research overview*[tiab] OR collaborative review*[tiab] OR collaborative overview*[tiab] OR systematic review*[tiab] OR technology assessment*[tiab] OR technology overview*[tiab] OR "Technology Assessment, Biomedical"[mh] OR HTA[tiab] OR HTAs[tiab] OR comparative efficacy[tiab] OR comparative effectiveness[tiab] OR outcomes research[tiab] OR indirect comparison*[tiab] OR ((indirect treatment[tiab] OR mixed-treatment[tiab]) AND comparison*[tiab]) OR Embase*[tiab] OR Cinahl*[tiab] OR systematic overview*[tiab] OR methodological overview*[tiab] OR methodologic overview*[tiab] OR methodological review*[tiab] OR methodologic review*[tiab] OR quantitative review*[tiab] OR quantitative overview*[tiab] OR quantitative synthes*[tiab] OR pooled analy*[tiab] OR Cochrane[tiab] OR Medline[tiab] OR Pubmed[tiab] OR Medlars[tiab] OR handsearch*[tiab] OR hand search*[tiab] OR meta-regression*[tiab] OR metaregression*[tiab] OR data synthes*[tiab] OR data extraction[tiab] OR data abstraction*[tiab] OR mantel haenszel[tiab] OR peto[tiab] OR der-simonian[tiab] OR dersimonian[tiab] OR fixed effect*[tiab] OR "Cochrane Database Syst Rev"[Journal:__jrid21711])) AND pharmaceutical) Filters: Humans; English
