# Supplementary material for: Comparative effectiveness and safety of pharmaceuticals assessed in observational studies compared with randomized controlled trials
Source: BMC Med. 2021 Dec 6;19:307. doi: 10.1186/s12916-021-02176-1 (PMC8647453; doi:10.1186/s12916-021-02176-1)
Supplement: Supplementary file 2 — Additional File 2:. Sensitivity Analyses [file 12916_2021_2176_MOESM2_ESM.docx]

**Sensitivity Analysis #1:**

The analysis was repeated with one endpoint from each review.

Criteria for selection of the endpoint if the review assessed more than one endpoint:

1. We ranked the endpoints by the most to least frequent across the reviews as listed below, and chose the endpoint highest on the frequency list whenever possible.
   1. Mortality/Survival
   2. Bleeding
   3. Stroke/TIA/Thromboembolic events
   4. Heart failure hospital admission/readmission,

Myocardial infarction,

Acute kidney injury/renal impairment

- 1. All other endpoints

1. If #1 did not result in the selection of a single endpoint, we chose the endpoint with the largest number of studies (RCTs and observational studies combined).
2. If #2 did not result in the selection of a single endpoint, we chose the endpoint with the narrower confidence interval for the ratio of observational/RCT effect estimates.

**Sensitivity Analysis #2:**

There were 26 studies that were included in more than one review. Therefore, the analysis was repeated ensuring that each study was included in only one review included in our analysis. The review with the greatest number of studies was chosen and included in this sensitivity analysis.
