# Supplementary material for: Comparative effectiveness and safety of pharmaceuticals assessed in observational studies compared with randomized controlled trials
Source: BMC Med. 2021 Dec 6;19:307. doi: 10.1186/s12916-021-02176-1 (PMC8647453; doi:10.1186/s12916-021-02176-1)
Supplement: Supplementary file 3 — Additional File 3:. Figures S1 & S2. Figure S1. Relative effect measures from observational studies versus corresponding relative effect measures from randomized controlled trials by outcome type. Figure S2. Relative effect measures from observational studies versus corresponding relative effect measures from randomized controlled trials by therapeutic area. [file 12916_2021_2176_MOESM3_ESM.docx]

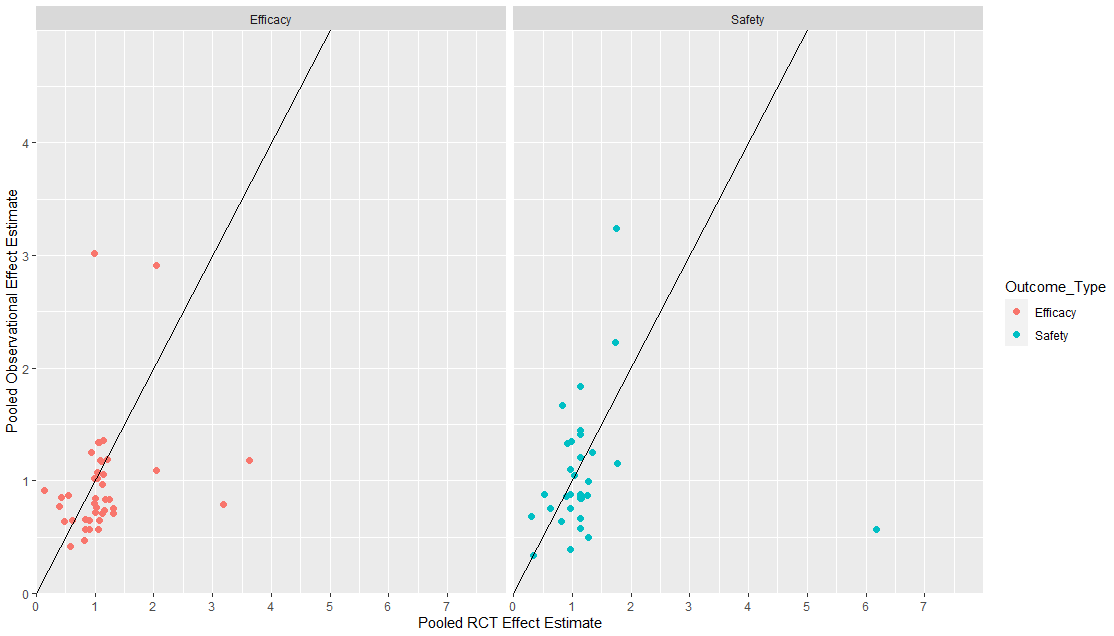


Figure S1. Relative effect measures (RR, OR, HR) from observational studies (y-axis) versus corresponding relative effect measures from randomized controlled trials (x-axis) by outcome type.


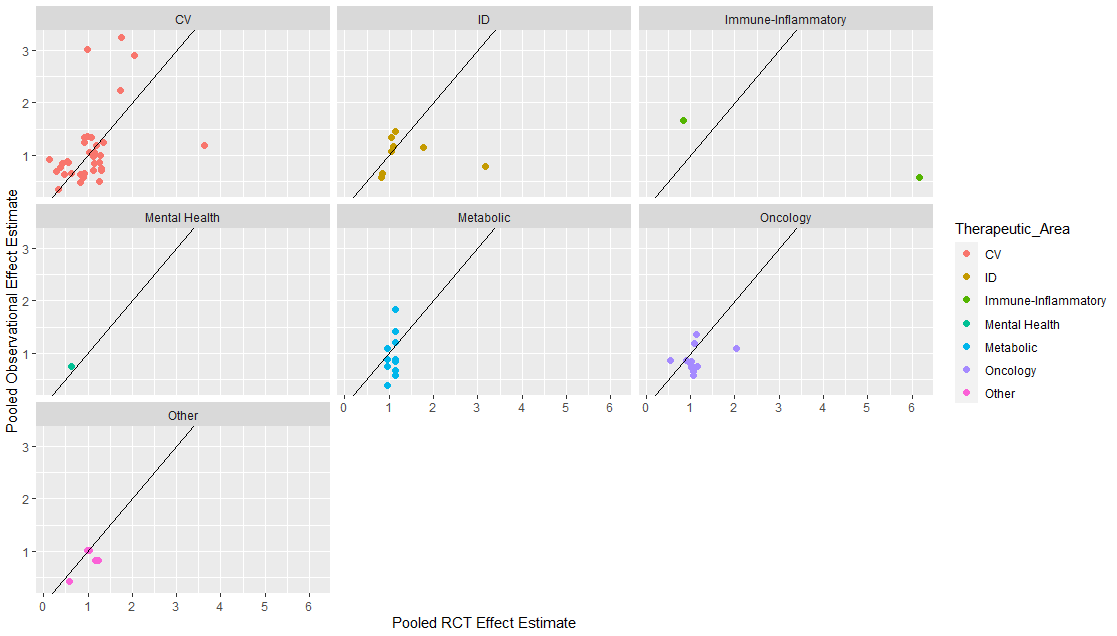


Figure S2. Relative effect measures (RR, OR, HR) from observational studies (y-axis) versus corresponding relative effect measures from randomized controlled trials (x-axis) by therapeutic area.
